# Food Webs in the Human Body: Linking Ecological Theory to Viral Dynamics

**DOI:** 10.1371/journal.pone.0048812

**Published:** 2012-11-14

**Authors:** Carmen Lía Murall, Kevin S. McCann, Chris T. Bauch

**Affiliations:** 1 Department of Integrative Biology, University of Guelph, Guelph, Ontario, Canada; 2 Department of Mathematics and Statistics, University of Guelph, Guelph, Ontario, Canada; Dalhousie University, Canada

## Abstract

The dynamics of in-host infections are central to predicting the progression of natural infections and the effectiveness of drugs or vaccines, however, they are not well understood. Here, we apply food web theory to in-host disease networks of the human body that are structured similarly to food web models that treat both predation and competition simultaneously. We show that in-host trade-offs, an under-studied aspect of disease ecology, are fundamental to understanding the outcomes of competing viral strains under differential immune responses. Further, and importantly, our analysis shows that the outcome of competition between virulent and non-virulent strains can be highly contingent on the abiotic conditions prevailing in the human body. These results suggest the alarming idea that even subtle behavioral changes that alter the human body (e.g. weight gain, smoking) may switch the environmental conditions in a manner that suddenly allows a virulent strain to dominate and replace less virulent strains. These ecological results therefore cast new light on the control of disease in the human body, and highlight the importance of longitudinal empirical studies across host variation gradients, as well as, of studies focused on delineating life history trade-offs within hosts.

## Introduction

The future of infectious disease control is threatened by the growing frequency of evolutionary responses of pathogens to antibiotics, anti-viral therapies, and vaccines [1], [2]. Some ‘imperfect’ or ‘leaky’ vaccines [3] allow the pathogen the opportunity to evolve, potentially leading to either increased virulence (e.g. Marek’s disease, [4]) or increased prevalence of non-target strains, known as strain replacement (e.g. 7-valent pneumococcal conjugated vaccine [5]). These challenges have led researchers to adopt new approaches that move beyond the basic biology of infections. Most notable are the studies of the kinetics [6], [7] and of the evolutionary biology of infections [8], [9]. These approaches distinguish themselves by describing interacting strains and immune cells as dynamical systems, and are used to understand persistence and virulence. This research, therefore, is closely aligned with classical food web research, which seeks to understand the persistence of whole ecological communities.

Though the need to untangle in-host ecological interactions is increasingly recognized [10]–[12], ecologically inspired empirical studies are not yet common, particularly for infectious diseases that affect humans. In contrast, ecology is part of the backbone of theoretical approaches to in-host studies because the original in-host models sprang from classic population ecology [13]. The analogy is that the immune system effector cells (e.g. cytotoxic T-cells, CTL, or B cells) target the invading parasite population in a similar fashion to predators consuming prey (e.g. CTL destroy infected cells). Consequently, most in-host models in the literature are Lotka-Volterra-like systems (for a review see [14]).

The analogy can be furthered to view the entire body as an ecological environment in which pathogens, host resources and immunity form networks of interacting populations that are analogous to food-webs [15]–[17]. In a rare example of this extended analogy, Pedersen and Fenton [16] broke down the entire in-host parasite-host system into community networks according to regions of the body, and argued cogently that further understanding in disease control would require the development of a strong multi-species approach. Similarly, Smith and Holt [18] emphasized the idea of the host as the environment for pathogen growth and competition. One approach to uniting these earlier efforts, and one taken in ecology, is to derive a theory for important sub-systems (modules) as a function of changing environmental conditions.

**Figure 1:**
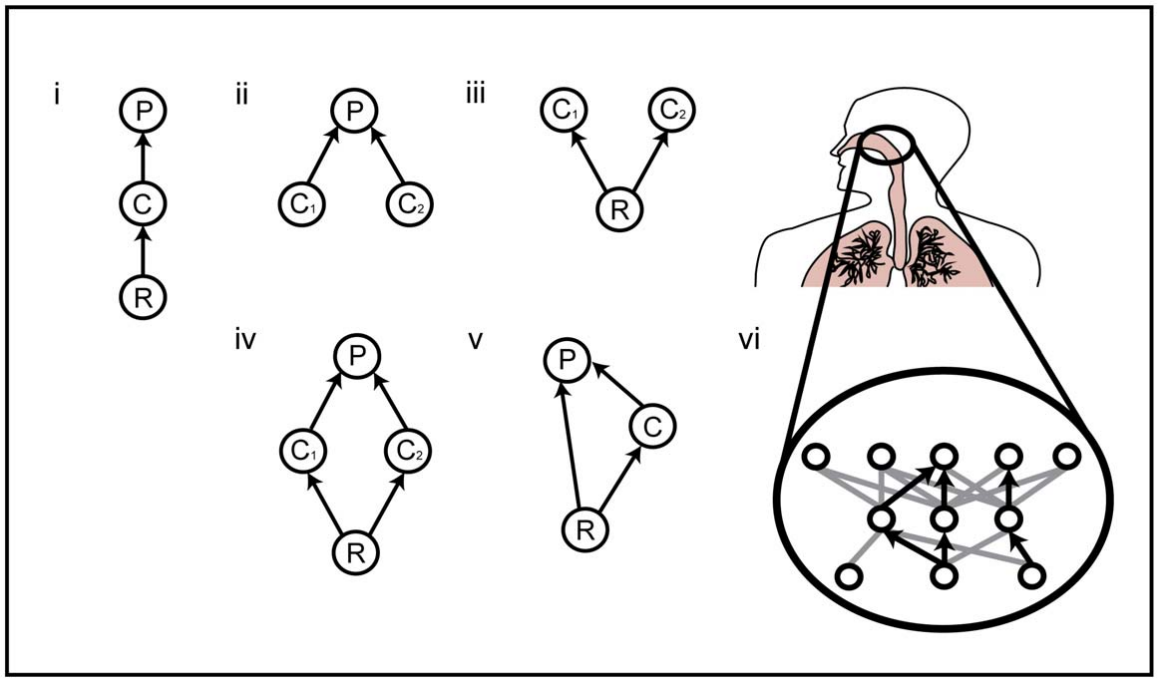
Common community modules in both free-living and in-host systems. P = predator (e.g. carnivore), C = competitor (e.g. herbivore) and R = resource (e.g. plant species). Modules: (i) Single-chain (ii) Apparent competition (iii) Resource competition (iv) Diamond (v) Intraguild predation, (vi) Modules are sub-webs of a larger web of all interacting host cells and coinfecting parasites.

Much recent food web research uses food web modules, multi-species extensions of pair-wise interactions between consumers and resources (Fig. 1). These modules, or “motifs” in network theory, are sub-networks used to probe the dynamical behavior of larger ecological communities [19] (see Box S1 for review of modular theory). Though modules (Fig. 1i–iv) are regularly found in infectious disease studies (e.g. immune-mediated apparent competition, [20], [21], Fig. 1ii, and model 3), surprisingly, a modular theory of in-host systems is not yet developed. Modular food web theory has found that species persistence depends on the topology of the web, as well as the strength of these interactions [22]. This suggests that to understand persistence, variation in disease burden, or strain dynamics in the human body, we need to identify the main players in the ecological network (e.g. which cells or cytokines fight a particular infection) as well as the relative strengths of the interactions.

Life histories of species in a community play an important role in food web dynamics. Interestingly, life history trade-offs often mediate the strength of interactions between species. As an example, let us consider species interacting in the diamond food web module (Fig. 1iv) , where the intermediate consumers (C_1_ and C_2_) are under both predation and competition pressure (Fig. 1iv). Ecologists have regularly found trade-offs between competitive strength (i.e. growth) and the ability to avoid predation, such that, fast growing, highly competitive organisms put little energy into defenses, while slow growing, weak competitors heavily invest energy into defense mechanisms. It is well known that this trade-off can yield coexistence [20]. Like their ecological counterparts, “in-host trade-offs” should arise because energy and time limit the replication and development of the parasite, and limit the production of immunity defense strategies.

While an important trade-off between virulence and transmission is thoroughly explored in disease research [14], examples of empirical in-host trade-off studies are surprisingly few, and we know of only one such virus study. De Paepe and Taddei (2006) found that the mortality rates of bacteriophages were positively correlated with multiplication rates, thus showing that the reproduction-and-survival trade-off also acts on viruses. They argued that the cost of having higher multiplication rates led to the production of more unstable virions because the thickness of the capsids and the density of the packed genomes were compromised [23]. Clearly, other in-host trade-offs ought to exist, and below we make theoretical arguments that suggest focused empirical research on in-host trade-offs can importantly delineate the abiotic conditions within the human body that drive either the dominance of virulent, or non-virulent, strains.

Finally, the application of drugs and vaccines clearly resonates with a major area of interest in food webs which is concerned with the implications of press (continuous) and pulse (discrete) perturbations on whole ecosystems [24], [25]. Drugs and vaccines effectively alter the strength of in-host web interactions by decreasing resource use or increasing pathogen visibility [26] and by boosting the ability of the immunity effector cells to quickly attack the infection, respectively. In an ecological sense, these control methods are strong perturbations, and it is interesting to note that they are often introduced without consideration of the environmental conditions and food web structure inside the host. A timely example of the introduction of vaccines without a clear picture of strain interactions and in-host ecology occurs in Human Papillomavirus (HPV). The strain-specific HPV vaccines target the two most virulent types (synonymous to ‘strain’), and they provide some cross-protection against a few antigenically similar types [27]. We considered how ecological understanding from the framework presented here could help explain HPV vaccine efficacy.

In what follows, we first revisit food web theory to show that changing environmental conditions modify the outcome of competition in predictable ways. We then extend this theory by considering the diamond food web module (Fig.1iv and model 2) within a disease framework. The diamond module ought to be common in hosts given that in localized regions of the body pathogens regularly share resources (cells) and often share a common predator (adaptive or innate immune responses [28]). With this viral dynamics food web module (model 2), we then look at how in-host life history trade-offs mediate competition and the disease burden between virulent and non-virulent strains across a gradient in host conditions. We end by analyzing the diamond and apparent competition modules (models 2 & 3) in different in-host environmental conditions, each constrained by empirically-estimated parameter sets and find that the results are remarkably consistent, which emphasizes the generality and plausibility of these results.

## Results

### Historical Results: Food Webs Across Changing Environmental Conditions

**Figure 2:**
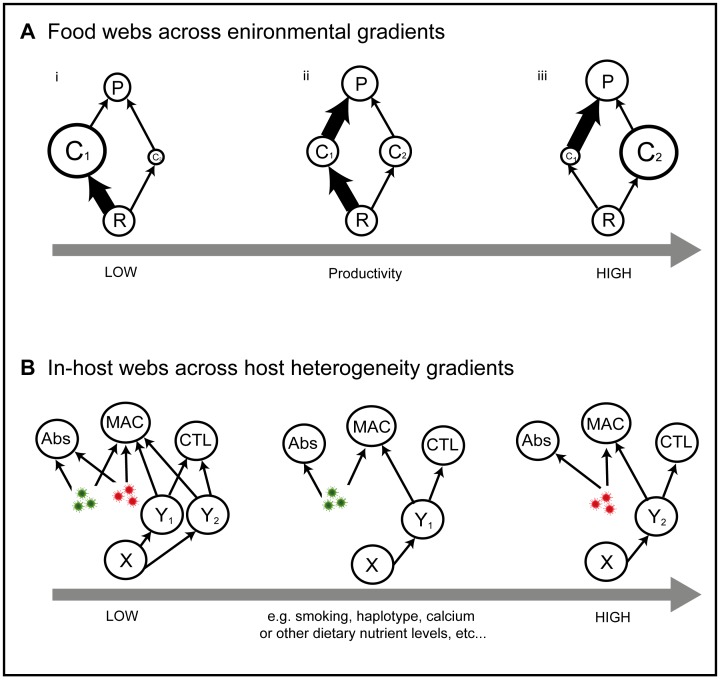
Variation between hosts as environmental gradients. The different weighted arrows represent the overall interaction strengths, and the various sized circles indicate relative densities of: shared resource, *R*, competitor species *i*, *C_i_*, top predator, *P*, antibodies, *Abs*, macrophages, *MAC*, cytotoxic T cells, *CTL* , uninfected cells, *X*, and infected cells by strain or species *i*, *Y_i_*. Similar to studies of abiotic environmental gradients studied in ecology (**A**), finding patterns of dynamic behaviours and outcomes across host heterogeneities (**B**) could greatly improve our understanding of variability in disease burden and what factors affect virulence and persistence.

Species coexistence, or dominance, depends greatly on environmental context. To illustrate this, we review a well-known result from ecological theory that considers a trade-off between growth and predation defense across a range of environmental productivity (Fig. 2A). For low productivity, the high growth species, *C_1_*, is able to suppress the common resource, *R*, to a point where the slow growing competitor, *C_2_*, has negative growth rates (i.e. it has lower R*, sensu [29], see Box S1). This leads to the dominance of the species with the fast growing strategy and the exclusion of the slow growing species, because, at low productivity, there is only enough energy to maintain a low density of predators (Fig. 2A.i). However, at intermediate productivity, both species coexist as predator densities are now elevated enough that the predator consumes the faster growing species *C_1_*, to an extent that allows the slower growing, well-defended species, *C_2_*, entry into the community (Fig. 2A.ii). At higher productivity still, the well-defended strategy dominates as predation increases to such an extent that the fast growing, highly consumed, species is decimated (Fig. 2A.iii).

In summary, life history trade-offs mediate the strength of the interactions between populations of the community. Here, the slower growing competitor is a weaker consumer of the common resource but is also weakly consumed by the predator (i.e. the weak chain; Fig. 2A.ii, R-C_2_-P chain), while the faster growing competitor is a stronger consumer of the common resource but also strongly consumed by the predator (i.e. the strong chain; Fig. 2A.ii, R-C_1_-P chain). This growth-defense trade-off, then, couples a weak food chain and a strong food chain. Therefore, under changing environmental conditions, trade-offs like these, mediate competitive outcomes in predictable ways (i.e. *context dependence*). This context dependent result pushes us to ask an interesting, if simple, question for disease ecology. Can we expect the individual abiotic conditions of the human body to also play a fundamental role in mediating the dominance, or not, of virulent disease strains? We examined this within a viral disease food web framework, with the hope that this gradient idea leads to predictable dynamical outcomes (Fig. 2B) as they do in free-living food webs (Fig. 2A).

### Disease Food Webs and Life History Trade Offs

**Figure 3:**
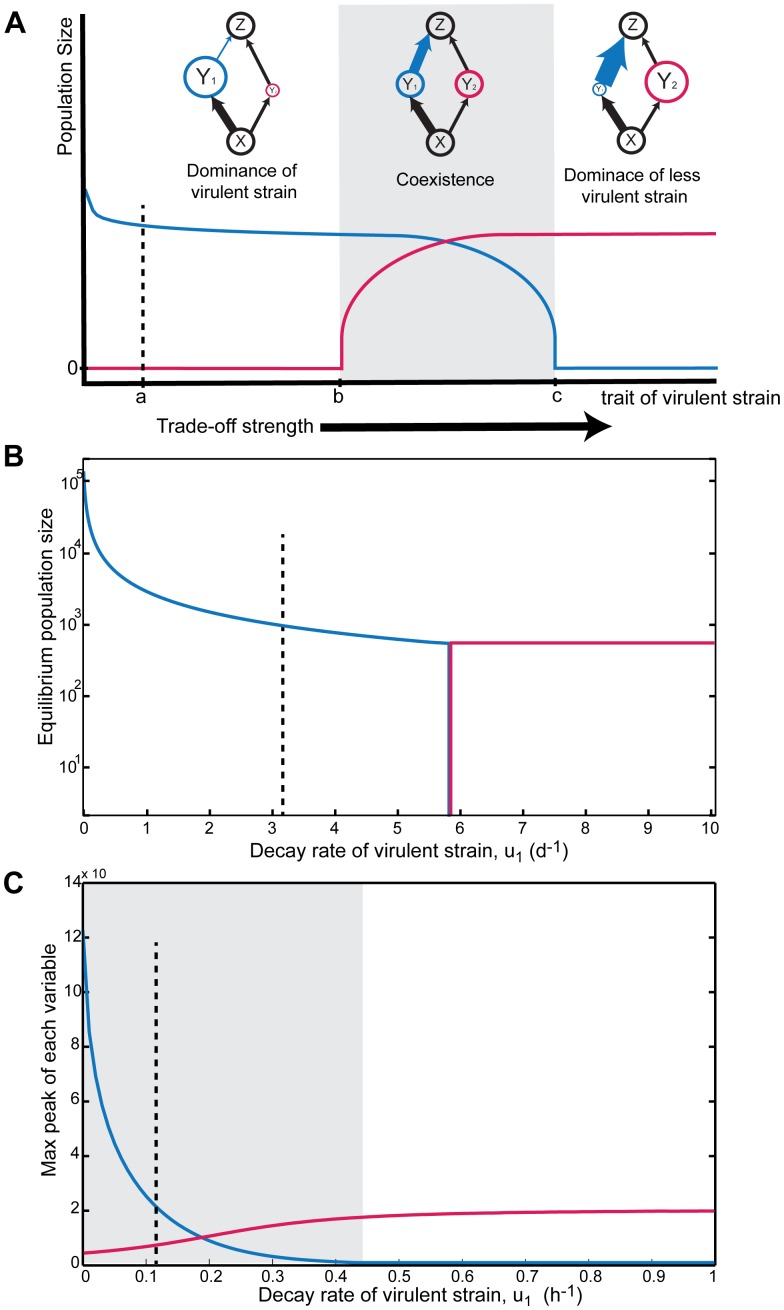
Effects of in-host trade-offs on dynamical outcomes. Viral loads of the virulent strain (strain 1; blue), and of the less virulent strain (strain 2; red). **A: Example bifurcation plot.** Replication-defense trade-off. As the cost of higher replication increases, transient coexistence becomes possible (area near the edge of shaded region). If this cost is raised even higher (less investment in immune defenses; blue link becomes stronger), then coexistence or dominance of the less virulent strain are possible. Mapped to each dynamical outcome is the corresponding module with its respective interaction strengths and relative densities. **Replication and decay trade-off in viral dynamics model:** ***Chronic and transient infections.*** HIV is plotted in **B** and Influenza A in **C.** The dashed lines indicate where both strains have identical decay rates (*u_1 = _u_2_*), which is where most conventional in-host models fall. However, without strain-specific measurements of viral decay rates we do not know where the system actually lies along these plots.

To explore how the strength of an in-host trade-off influences competition between strains, we performed simple numerical experiments by varying a trait involved in a given trade-off such as the mortality rate (decay rate) of the virulent strain and followed a given property of the dynamics (e.g. the equilibrium densities) of the strains as a response variable (see Fig. 3A for a schematic). We focused on the reproduction-and-decay trade-off [23], though results of another in-host trade-off is in the Supplementary Material (Text S1). In the parameter space considered, the full (1) and diamond models (2) gave nearly identical results (not shown) only the diamond model (2) does not output viral loads. However, a more formal analysis of these two models would be needed to see if and when the similarity in dynamical behaviour between models breaks down. This implies that the two-strain viral dynamics model behaves like a diamond module, therefore, consideration of the diamond module ecological literature should be of interest to those investigating viral strain competition. The plots presented here are of the full model (1) because discussion of viral loads, the main measurable quantity of infections, is best for empirical comparisons.

As expected from modular theory, the results suggest that trade-offs may be extremely powerful in mediating the dominance or coexistence of virulent and non-virulent strains (or less virulent), regardless of whether the infection was chronic (Fig. 3B) or acute (Fig. 3C). Clearly, there is little cost to higher reproduction rates until the decay rate of the virulent strain is greater than the less virulent strain (i.e. to the right of the dotted lines in Fig. 3B and C). Varying the strength of the trade-off gave a common sequence of events: virulent dominance, coexistence, and non-virulent dominance. In the case of HIV, the dominance of the strains switches as the strength of the trade-off changes without passing through coexistence (Fig. 3B; no grey region) but in the acute infection case, the coexistence region comes first and then enters into non-virulent dominance (Fig. 3C). See Supplementary Material for conditions that give coexistence (Text S1, Fig. S1 and S2) and for another example of this sequence of outcome events as the strength of the reproduction-and-lytic-effect trade-off is increased (Fig. S3). These results, then, are consistent across different viral empirical parameter sets. Also, given that the strength of life history trade-offs alter coexistence, it becomes interesting to also consider how life history trade-offs interact with changing host conditions to alter coexistence.

### Disease Food Webs and Changing Host Environmental Conditions

**Figure 4:**
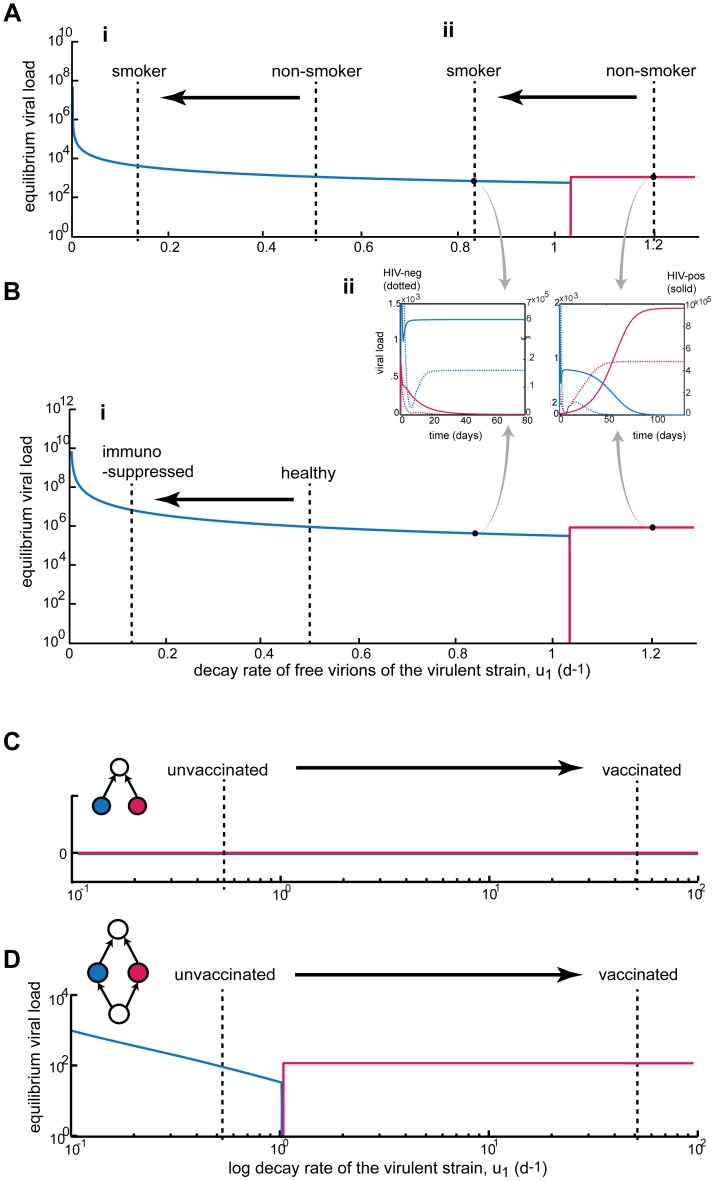
Immune deficiency and vaccination in HPV. Viral load of HPV-16 (blue), viral load of non-vaccine HPV type (red). **A: Immunocompetent CTL response.** (**i**) Smoking impairs the humoral response which shifts the system away from the bifurcation, resulting in higher viral load and thus higher disease burden. (**ii**) Hypothetical scenario: Smoking changes the strain dominance structure, by weakening the strength of the natural trade-off, so that the more virulent strain is dominant in the smoker. Epidemiologically, some strains would be more prevalent in smokers than in non-smokers, and their viral loads would be significantly higher. **B: HIV-positive hosts with HPV infection.** Over the entire trade-off axis the viral loads are higher (than in A) due to the depletion of the CTL population by HIV. (**i**) Similar to A.i, the effect of the simultaneous suppression of the humoral system is augmented by the in-host trade-off. (**ii**) Example time series at u_1_ = 0.83 and u_1_ = 1.2. Another dynamical consequence of CTL depletion by HIV is that HPV types coexist longer inside the host (differences in time till exclusion of one type). Compare transients of dotted (immunocompetent) vs. solid (immunocompromised) curves. **Vaccination. C:** If stochasticity near zero is considered, then vaccination in the immune-mediated apparent competition module leads to the clearance of both strains, i.e. cross-protection (here the curves represent infected cells of the HPV types). **D:** In contrast, the diamond module, with shared resources, behaves differently. By increasing the strength of the trade-off, the vaccine changes the conditions to favor the less virulent strain.

We considered how smoking, a host behavior that changes abiotic in-host environments, can be a potential environmental gradient across which in-host disease ecology can vary. Figure 4 A.i shows how smoking can affect the outcome of hosts infected with two HPV types. The non-smoker line indicates where the virion decay rate of HPV-16, *u_1_*, is within a non-smoker. Because smoking impairs the ability of antibodies to neutralize free virions [30], then the smoker host is necessarily to the left of the non-smoker (the exact quantity is not known only the direction) and so the smoker experiences higher viral loads because more virions are able to infect cells (Fig. 4 A.i). The result in Fig. 4 A.i was fairly robust because the qualitative results were not affected by including either impaired CTL attack or suppression of LC, but differential CTL killing rates did. For example, if the CTL killing rate of HPV-16 infected cells is more impaired by smoking (i.e. *p_1_* < *p_2_*), then the bifurcation plot is shifted to the right (not shown). Biologically this means that HPV-16 will dominate at a higher viral load because as the difference between the CTL killing rates against each strain increases, the biologically reasonable region is closer to the steepest region of the viral load of HPV-16 (i.e. closer to the origin). This implies that smokers will have a much higher viral load than is demonstrated in Fig. 4 A.i.

We considered another informative example, where we assumed that the conditions in healthy hosts favored the less virulent strain. Not surprisingly, the less virulent strain dominates the non-smoker (Fig. 4 A.ii), however, if the same level of smoking impairment is included we find that the system is now shifted to a completely different equilibrium, where the more virulent strain dominates the infection. Consequently, in such a case, smokers can experience a dramatically different outcome in that they have infections dominated by virulent strains. Alarmingly, the result of a simple human behavior pushes the host to an entirely different equilibrium that is potentially life threatening.

Finally, we considered another example of immunosuppression: HIV infection. Here, the depletion of CTL lead to higher HPV viral loads (a consequence of a larger number of infected cells; Fig. 4B), which corresponds to what is known about HIV-positive HPV infected hosts [31]. This high viral load can be increased further with the trade-off (Fig. 4B.i), again demonstrating that dynamics and in-host trade-offs could help explain clinical results and variation of disease burden across hosts. Finally, this immune suppression by CTL depletion gave longer transients (Fig. 4B.ii dotted vs. solid line). In the parameter range considered, on average the HIV-positive environment took 92 days longer for the exclusion of one of the types (range was 9–350 days longer to exclusion, where the longer transients were near the bifurcation). Therefore, ecologically HPV types are transiently coexisting for longer and so, clinically, more HPV types are more likely to be detected than in immunocompetent infections where exclusion happens faster.

### Disease Food Webs and Disturbances: Drugs and Vaccines

Currently cross-reactivity is the only known form of HPV type-type interaction. The HPV community then is assuming that the underlying in-host web is an apparent competition module (Fig. 1ii). When we considered vaccination in this apparent competition module (model 3), the in-host trade-off (varying u_1_) had a similar qualitative effect, i.e. switching from Y_1_-wins to Y_2_-wins equilibrium with the same bifurcation. Nevertheless, the infected cell equilibria are close to zero (< 1) so the oscillations of the transients drive them to crash, and subsequently the very large CTL population also crashes. Therefore, if you consider stochastic effects close to zero and very strong overshoot properties of the transients, the vaccine case in the apparent competition module corresponds to vaccine clearance of both types via cross-protection (Fig. 4C). However, should this cross-reactivity only assumption be incorrect and instead the web structure is the diamond model (which we suspect it might be), then the vaccine CTL do not suppress the two types as much (Fig. 4D). Thus increasing the antibody neutralization rates to vaccine levels against HPV-16 does change the outcome of the system, where now the non-vaccine type has a higher viral load than in the unvaccinated host (Fig. 4D). Therefore, on account of resource competition, the slower reproducing strain is not completely cleared by the cross-reactive vaccine and, though at low levels, now dominates the system (a phenomenon called “type replacement”). This demonstrates that the underlying web matters and that the unwanted outcome of competitive release could be amplified because the vaccine changes the in-host trade-off. It is imperative to look for evidence of resource competition between high-risk HPV types, regardless of their antigenic similarities.

## Discussion

Here, we have extended ecological theory to show that host abiotic environment coupled to life history trade-offs may play a fundamental, but underappreciated, role in the dynamics of infectious diseases. Specifically, we have shown that even modest differences in host environment can significantly change disease burden. Similarly, human behavior that alters environmental conditions (e.g. smoking) has the potential to flip the in-host ecosystem from non-virulent viral strain dominance to one dominated by virulent strains. Interestingly, patients are asked to stop smoking to help clear HPV infections, and this in-host community dynamics and trade-offs framework helps interpret why this may often work. Similarly, the results of the HIV-positive environment captured empirically known features of higher viral loads and more coinfection with non-HPV-16 types [32]. Our environmental gradient analogy, then, could aid in building a more mechanistic understanding of variation in disease burden and clearance across different patients.

The application of food web theory to the in-host environment can be further justified when the role of pathogens in classical ecological food webs is considered. The parasitic strategy is fundamentally a consumer strategy, whereby fluxes of energy and biomass flow from the host to the parasite [33]. The in-host environment, then, is not a completely separate system from the larger food web. Consequently, we believe, viruses also participate in this biomass and energy loss, though they themselves are not cellular organisms. Because viruses cannot perform their own metabolic processes, they hijack the host cell’s metabolic products in order to replicate themselves. This is a redirection of energy that the virus now uses, not the host. The immune systems’ role is to break the link between parasite and host and stop the energy loss. The immune system does not directly get energy or biomass from the parasite but it indirectly benefits by the depletion of the parasite population because the host has more resources to contribute to the immune system.

Ultimately, however, what food web theory offers most to infectious disease studies is a community approach that compliments the conventional reductionist approach. For instance, the concept of meta-populations has aided developing epidemiological theories and models of infectious diseases [34]. A true extension of this work will be to consider populations of hosts as *meta-communities*. This attempt to connect in-host ecology and between-host ecology should help us understand how these two ecological stages affect the evolution of infectious diseases [35].

We show here that if HPV coinfection modules are more complex than currently expected, then the cross-protection of the vaccines may not be as strong as expected for some types. Thus the underlying module of the in-host community could affect vaccination outcome, and indeed, a couple of types have been identified as types that could potentially benefit from the vaccine, namely HPV-33 [36], and HPV-52 [37]. Note that our results are tempered by the fact that trade-offs experienced by HPV have not been looked for or found. We also found that a dynamical consequence of HIV infection was longer transient coexistence of HPV types. This ecological result could help explain why HIV-positive patients have more multiple infections [32]. Since HPV types regularly coexist in the same hosts, this virus is an interesting model to study the mechanisms of strain coexistence and in-host trade-offs experienced by viruses.

For simplicity, we assumed that the strains do not evolve or change phenotypes (e.g. shedding of an antigenic coat) during the course of the infection. Nonetheless, fast evolving infectious diseases still fit this framework. In fact, their rapid evolution exploits life history trade-offs by changing the relative costs of advantageous traits. One can envision that the evolutionary change allows the system to move across these plots during the course of an infection. Since rapid evolutionary changes affect the important rates of the system which determine the interaction strengths, they effectively alter dynamical outcomes. Life history trade-offs, then, serve as a link between evolutionary changes and the ecology of the infection.

Strains can be distinguished by a suite of traits, yet strains in most current models usually differ only by their replication or infection rates. Thus, a priori, these models tend to exclude cases in which the life histories of the strains differ. As shown here, differences in traits other than replication rates can have measurable effects. Consequently, reasonable estimates of strain-specific mortality rates, burst sizes, immunity evasion rates, etc., in conjunction with knowing what in-host trade-offs exist, should allow us to better predict and understand the outcomes of coinfections.

Taxonomic studies, distinguish strains by their genetic differences, and attempt to piece together the evolutionary history of their genes (example [38]). A potentially fruitful avenue of study would be to find the ecological context in which traits are favored by tying these genetic variations to their role in life history and in-host trade-offs, while also searching for the costs of traits and not just focusing on their advantages.

For example, HPV types are either of high- or low-oncogenic risk. This depends on their host cell transformation properties, where their early proteins E6 and E7 work together to extend the life of the host cell (prevent apoptosis) and stimulate cell cycle progression [39]. This strategy appears to be advantageous for high-risk types, because, they increase: a) the number of infected cells without having to burst from one cell and then find another cell to infect, and b) the viral production per infected cell. Though, to be clear, the malignization of the host cell (which usually happens several years later) is not what increases viral fitness. Even though these traits appear advantageous, we should also ask: what are the low-risk types good at that would explain why they can coexist with high-risk types? Also, is there a cost to these cell transformative traits of high-risk types? This exemplifies that our current focus on virulent forms limits our understanding of in-host trade-offs and dynamics.

Future in-host models of HPV should include several features ours did not. For example, considering the spatial nature of HPV infection could be used to address other interesting questions, such as, why is the distribution of HPV types in normal mucosa different than in cancer tissue? Can ecological aspects of the in-host environment play a role (e.g. are types being competitively excluded over time)? Though the spatial ecology of HPV was not considered here, the importance of space cannot be understated. Host resources might be clumped or sparse, and the distribution of different cell types is also heterogeneous, therefore, other modeling methods, such as partial differential equations or individual-based-models, may be more appropriate for capturing these interactions and local spatial effects [40]. Spatial heterogeneity can change the strengths of the interactions that in well-mixed environments are strong, and the spatial coupling of more weak interactions can lead to more stable dynamics and less oscillations [41]. Studies of HPV in-host ecology in future models could also help elucidate other HPV debates that could be related to resource competition between types (e.g. do types cluster together? [42] Or are lesions formed by only one HPV type? [43]). Our model was a first and course attempt at addressing some of the many interesting questions in HPV that could be tackled using this in-host community ecology approach.

The models used here were kept simple in order to maintain explanatory power for conceptual development. Changes to our assumptions would lead to interesting extensions of this work. For example, we assume the in-host environment is held constant over the course of the infection, and yet, non-constant environments would be very interesting to consider. Issues of drug non-compliance, self-medication or habits that cycle could impact in-host dynamics, much like environmental perturbations or seasonal/periodic changes ecologists study. Another example of an assumption change, would be to include coinfection of cells, which not only would affect strain-interactions [44] but could also be considered a change in community module, one akin to the intraguild predation module [45] found in free-living systems (Fig. 1v). Finally, an important change to these assumptions is finding more realistic and appropriate forms for the rates presented here. For instance, in ecology, modes of predation have been well characterized (functional and numerical responses) but, in-host dynamic studies are yet to fully capture and synthesize the immune cell-pathogen interaction types. Excitingly, the field of mathematical immunology is growing rapidly and, they are finding features that depart from classic predator-prey interactions [46].

We propose two in-host trade-offs to be sought after in future work. First, the reproduction-and-predation-resistance trade-off found in free living organisms, can be envisioned as a trade-off between reproduction-and-immune-evasion, where there is a choice between allocating energy and resources to efficient replication or to immune evasion methods (e.g. anti-interferon or down-regulation of TLR) or to other mechanisms that interfere with the immune system. Second, a trade-off between resource-use-and-immune-evasion would be analogous to the predation risk of foraging. In viruses, the more time spent inside host cells (even if reproducing at low levels) increases the likelihood of being detected by the immune system. These trade-offs might help us understand the context under which immune evasion strategies evolve.

Though there are features unique to in-host environments [46], it is exciting to explore how they fit within the ecological stage in which these systems unfold. Studies using evolutionary ecology methods that look for in-host trade-offs in viral families infecting humans could lead to a powerful understanding of how strains interact. This type of viral ecology research could also help motivate our decisions as to which interactions to strengthen or weaken artificially using drugs or vaccines, as well as, inform as to why some attempts fail.

With the development of more sophisticated molecular techniques, probing in-host dynamics is increasingly more feasible and studying the in-host environment as a web of interacting populations is imminent. Studies that measure multiple populations longitudinally are crucial for grounding in-host theory. We suggest that comparisons of in-host dynamics across a range of hosts of a potential gradient (Fig. 2B) will accelerate our understanding of disease burden and clearance, and will be fundamental in our development of an ecological theory of the human body. This in-host web theory coupled to a more static molecular biology view of these infections will enrich and bring together the studies of kinetics and evolutionary biology of infections, while proving to be of enormous power in disease control.

## Methods

### Viral Food Web Models

The two-strain viral dynamics model with cross-reactive CTL immunity[13], [47] is
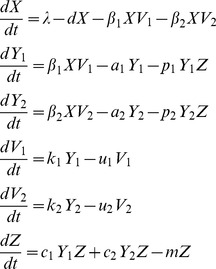
(1)


The uninfected cells, *X*, become infected cells, *Y_i_*, by contact with free virions of strain *i*, *V_i_*, and infected cells are killed by CTLs, *Z,* at a rate of *p_i_*. The uninfected cells are born and die at constant rates, *λ* and *d* respectively. Infected cells become infected, *β_i_^’^*, and die at a rate caused by the virus, *a_i_* (this equals *d* if the virus is non-lytic). Free virions are produced by the infected cells by rate *k_i_* and are cleared at a rate *u_i_* either due to decay, mucosal flushing or antibody neutralization. The CTL population grows at a rate proportional to the infected cell population, *c_i_Y_i_Z,* and the CTLs have a constant death rate, *m*.

In order to reduce this model to be more manageable we assumed the free virion variables, *V_1_* and *V_2_*, reach steady state because their dynamics are much more rapid than the other variables [44]. This reduction gives a viral dynamics model that is analogous to the diamond module in food webs
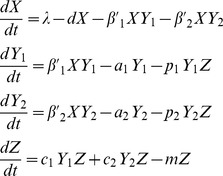
(2)where 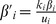. Also, model 2 can be modified to represent the apparent competition module [20] by assuming the resource, *X*, is constant. Therefore the infected cells, *Y_i_*, grow at a rate, 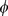 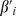, which is affected by the free virus population parameters. This gives,
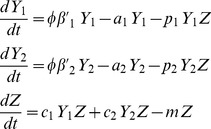
(3)where 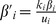. We used this immune-mediated apparent competition module for comparison to the diamond.

### Parameters

Parameter estimates were taken from the literature. HIV: *λ = 397 cells•day^−1^*, *d = 0.3 day^−1^*, *β_1_* = *β_2_* = *0.001 day^−1^*, *k_1_* = *1114 virions•cell^−1^•day^−1^*, *k_2_ = 600 virions•cell^−1^•day^−1^*, *u_2_ = 3.12 day^−1^*, *a_1_ = a_2_ = 0.76 day^−1^* all from[48]. Influenza A: *β_1 = _β_2 = _7.5×10^−7^ cells•hr^−1^, k_1 = _0.098 hr^−1^, k_2 = _0.064 hr^−1^, u_2 = _0.105 hr^−1^, a_1 = _a_2 = _0.066 hr^−1^* all from [7]. HPV: *λ = 36000 cells•day^−1^* [*49*] *, d = 0.048 day^−1^* [*50*] *, β_1 = _β_1 = _0.0067 day^−1^* [*51*] *, k_1 = _100 virions•cell^−1^•day^−1^ , k_2 = _50 virions•cell^−1^•day^−1^* [52], *u_2 = _0.52 day^−1^* [23], and since HPV is a non-lytic virus *d = a_1_* = *a_2_* = *0.048 day^−1^* [50].

Immunity against HIV: *p_1_ = p_2_ = 1 day^−1^* [53], *c_1_ = c_2_ = 0.3 day^−1^* [54], *m = 0.5 day^−1^* [55].

Immunity against HPV: (i) Immunocompetent: *p_1_ = p_2_ = 1 day^−1^* [53], *m = 0.5 day^−1^* [55], and because HPV is a poor natural immunogen and immunity against HPV types is cross-reactive at best *c_1_ = 0.1* and *c_2_ = 0.05 day^−1^* [56]. (ii) Immunodeficient (HIV-positive): *p_i_* and *c_i_* decreased 100 fold, while *m = 5*. (iii) Vaccine: *p_i_* and *c_i_* increased 100 fold, while m is same as in (i).

### Numerical Experiments

In order to unfold a trade-off, we assumed that the two strains were identical, except that the virulent strain had a higher reproduction rate. Thus, our experiment varies the cost (e.g. less investment in defenses or higher decay rate of the virulent strain) associated with having a higher reproduction rate (schematic Fig. 3A). Note that we start this experiment with the virulent form experiencing a low cost (to the left of the dotted line, *a*, in Fig. 3A) and end with a significantly higher cost (past *b* and *c*) where now the immunity attack rate (or decay rate) is much higher than its competitor. We ran this analysis on the two-strain viral dynamics model [13] (model 1 and 2) with the replication and decay trade-off for HIV and for HPV, using parameter estimates from in-host data in the literature. To our knowledge, we are the first to apply this model to HPV and to compile an in-host parameter set. For chronic infections, the stable viral loads (that are reached after some transient time) were plotted for various values of the decay rate of the virulent strain (Fig. 3B). To consider transient acute infections that do not reach equilibrium, we used a model specific for Influenza [7], and instead plotted the maximum peaks in viral load as a response variable (Fig. 3C).

To investigate the potential role of individual host conditions, we explored how the behavior of smoking can affect HPV type interactions. In HPV, smoking is known to decrease the strength of the CTL response [57], decrease the antibody response [30], [58], and is associated with lower numbers of intraepithelial Langerhans’ cells (LC) in the cervix [59]. These biological implications of smoking are specifically embedded in the two-strain diamond model (model 2) by reducing the parameters, *p_i_* and *u_i,_* which mimic impaired CTL and antibodies responses in the model [13]. We also considered the compound effect of impaired CTL attack (lower *p_i_*) and the suppression of LC (lower *c_i_*). The effect of HIV coinfection with HPV was considered by similarly lowering CTL response and increasing its natural death rate (higher *m*) due to HIV’s lytic activity.

We investigated how the introduction of the HPV vaccine would affect a coinfection in-host community that experiences a reproduction-decay trade-off. The vaccine targeted HPV-16 (strain 1) and weakly cross-protected against a related non-targeted HPV type (strain 2). Since the vaccine Gardasil© boosts antibody response by 100 fold [60], we considered this antibody increase in the free virus decay rate of HPV-16 (antibody neutralization is implicit in *u_i_*) in two modules, the diamond (with shared resource; model 2) and in the immune-mediated apparent competition module (without shared resource; model 3).

## Supporting Information

Figure S1 **Phase-plane cases.** **A: Competitive exclusion.** (**i** and **ii**) The black dots represent the equilibrium solution of each subsystem (i.e. where *X, Y_i_, Z* can exist together). The winner is determined by invasion criteria, such that if the isocline of strain *i* is above the equilibrium of the subsystem with strain *j*, then strain *i* can invade but strain *j* cannot. Strain *i* then, is the winner and its subsystem equilibrium is an attractor. **B: Coexistence.** Here the isoclines cross inside the two subsystem curves and both strains can invade, thus the interior equilibrium is stable. Finally, **C:** **Priority Effects.** The interior equilibrium is unstable therefore the initial conditions determine who wins.
(TIF)

Figure S2 **The reproduction and decay trade-off in HPV with matching phase-planes**. No coexistence. Plots (**i**) and (**iii**) represent the phase-planes before and after the bifurcation, respectively, and plot (**ii**) is at the bifurcation. *Parameter estimates.* HPV: *λ = 36000 cells•day^−1^* [49], *d = 0.048 day^−1^* [50], *β_1 = _β_1 = _0.0067 day^−1^* [51], *k_1 = _100 virions•cell^−1^•day^−1^* , *k_2 = _50 virions•cell^−1^•day^−1^* [52], *u_2 = _0.52 day^−1^* [23], and since HPV is a non-lytic virus *d = a_1 = _a_2 = _0.048 day^−1^* [50]. Immunity: *p_1 = _p_2 = _1 day^−1^* [53], *m = 0.01 day^−1^* [56], *c_1 = _c_2 = _0.1 day^−1^* [44]
(TIF)

Figure S3 **The reproduction and lytic effect trade-off in HPV allows for coexistence**. Plots (**i**) and (**v**) are before and after the bifurcations, (**ii**) and (**iv**) are at the bifurcations, and (**iii**) is stable coexistence. *Parameter estimates.* HPV: *λ = 36000 cells•day^−1^* [49], *d = 0.048 day^−1^* [50], *β_1 = _β_1 = _0.0067 day^−1^* [51], *k_1 = _100 virions•cell^−1^•day^−1^* , *k_2 = _50 virions•cell^−1^•day^−1^* [52], *u_2 = _0.52 day^−1^* [23], and since HPV is a non-lytic virus *d = a_1 = _a_2 = _0.048 day^−1^* [50]. Immunity: *p_1 = _p_2 = _1 day^−1^* [53], *c_1 = _c_2 = _0.1 day^−1^* [44], *m = 0.5 day^−1^* [55].
(TIF)

Text S1 **Conditions for Coexistence inside a Host.**
(PDF)

Box S1 **Using modules to understand community dynamics.**
(PDF)

## References

[1] Williams PD, Day T (2008) Epidemiological and evolutionary consequences of targeted vaccination. Molecular ecology 17: 485–499 doi:10.1111/j.1365-294X.2007.03418.x. 18173510 10.1111/j.1365-294X.2007.03418.x

[2] Andersson DI, Hughes D (2010) Antibiotic resistance and its cost: is it possible to reverse resistance? Nature reviews Microbiology 8: 260–271 doi:10.1038/nrmicro2319. 20208551 10.1038/nrmicro2319

[3] Gandon S, Mackinnon M, Nee S, Read A (2003) Imperfect vaccination: some epidemiological and evolutionary consequences. Proceedings of the Royal Society B: Biological Sciences 270: 1129–1136 doi:10.1098/rspb.2003.2370. 12816650 10.1098/rspb.2003.2370 PMC1691350

[4] Nair V (2005) Evolution of Marek’s disease – a paradigm for incessant race between the pathogen and the host. Veterinary journal 170: 175–183 doi:10.1016/j.tvjl.2004.05.009. 10.1016/j.tvjl.2004.05.009 16129338

[5] Greenberg D (2009) The Shifting Dynamics of Pneumococcal Invasive Disease after the Introduction of the Pneumococcal 7-Valent Conjugated Vaccine?: Toward the New Pneumococcal Conjugated Vaccines. Clinical Infectious Diseases 49: 213–215. 19508164 10.1086/599828

[6] Perelson AS (2002) Modelling viral and immune system dynamics. Nature reviews Immunology 2: 28–36 doi:10.1038/nri700. 10.1038/nri700 11905835

[7] Beauchemin CAA, McSharry JJ, Drusano GL, Nguyen JT, Went GT, et al (2008) Modeling amantadine treatment of influenza A virus in vitro. Journal of theoretical biology 254: 439–451 doi:10.1016/j.jtbi.2008.05.031. 18653201 10.1016/j.jtbi.2008.05.031 PMC2663526

[8] Gilchrist MA, Coombs D (2006) Evolution of virulence: interdependence, constraints, and selection using nested models. Theoretical population biology 69: 145–153 doi:10.1016/j.tpb.2005.07.002. 16198387 10.1016/j.tpb.2005.07.002

[9] Day T, Galvani A, Struchiner C, Gumel A (2008) The evolutionary consequences of vaccination. Vaccine 26: C1–C3 doi:10.1016/j.vaccine.2008.02.006. 18773533 10.1016/j.vaccine.2008.02.006

[10] Mideo N, Barclay VC, Chan BHK, Savill NJ, Read AF, et al (2008) Understanding and predicting strain-specific patterns of pathogenesis in the rodent malaria Plasmodium chabaudi. The American naturalist 172: 214–238 doi:10.1086/591684. 10.1086/591684 18834302

[11] Levin B (2001) How can we predict the ecologic impact of an antimicrobial: the opinions of a population and evolutionary biologist. Clinical Microbiology and Infection 7: 24–28. 10.1046/j.1469-0691.2001.00070.x 11990679

[12] Dennehy JJ, Friedenberg N a, Holt RD, Turner PE (2006) Viral ecology and the maintenance of novel host use. The American Naturalist 167: 429–439. 10.1086/499381 16673350

[13] Nowak MA, May RM (2000) Viral Dynamics: mathematical principles of immunology and virology. Oxford University Press. p.

[14] Alizon S, Hurford A, Mideo N, Van Baalen M (2009) Virulence evolution and the trade-off hypothesis: history, current state of affairs and the future. Journal of evolutionary biology 22: 245–259 doi:10.1111/j.1420-9101.2008.01658.x. 19196383 10.1111/j.1420-9101.2008.01658.x

[15] Frank SA (2002) Immunology and Evolution of Infectious Diseases. NJ: Princeton University Press. p. 20821852

[16] Pedersen AB, Fenton A (2007) Emphasizing the ecology in parasite community ecology. Trends in ecology & evolution 22: 133–139 doi:10.1016/j.tree.2006.11.005. 17137676 10.1016/j.tree.2006.11.005

[17] Fenton A, Perkins SE (2010) Applying predator-prey theory to modelling immune-mediated, within-host interspecific parasite interactions. Parasitology 137: 1027–1038 doi:10.1017/S0031182009991788. 20152061 10.1017/S0031182009991788

[18] Smith VH, Holt RD (1996) Resource competition and within-host disease dynamics. Trends in ecology & evolution 11: 386–389. 21237891 10.1016/0169-5347(96)20067-9

[19] Holt RD (1997) Community modules. In: Brown CG& VK, editor. Multitrophic inter- actions in terrestrial ecosystems, 36th Symposium of the British Ecological Society. London, UK: Blackwell Science Ltd. Ives.

[20] Holt RD (1977) Predation, apparent competition, and the structure of prey communities. Theoretical Population Biology 12: 197–229. 929457 10.1016/0040-5809(77)90042-9

[21] Mideo N (2009) Parasite adaptations to within-host competition. Trends in parasitology 25: 261–268 doi:10.1016/j.pt.2009.03.001. 19409846 10.1016/j.pt.2009.03.001

[22] Wootton JT, Emmerson M (2005) Measurement of Interaction Strength in Nature. Annual Review of Ecology, Evolution and Systematics 36: 419–444 doi:10.1146/annurev.ecolsys.36.091704.175535.

[23] De Paepe M, Taddei F (2006) Viruses’ life history: towards a mechanistic basis of a trade-off between survival and reproduction among phages. PLoS Biology 4: e193 doi:10.1371/journal.pbio.0040193. 16756387 10.1371/journal.pbio.0040193 PMC1475768

[24] Bender E, Case T, Gilpin M (1984) Perturbation experiments in community ecology: theory and practice. Ecology 65: 1–13.

[25] O’Gorman EJ, Emmerson MC (2009) Perturbations to trophic interactions and the stability of complex food webs. Proceedings of the National Academy of Sciences of the United States of America 106: 13393–13398 doi:10.1073/pnas.0903682106. 19666606 10.1073/pnas.0903682106 PMC2726361

[26] Colijn C, Cohen T, Fraser C, Hanage W, Goldstein E, et al (2010) What is the mechanism for persistent coexistence of drug-susceptible and drug-resistant strains of Streptococcus pneumoniae? Journal of the Royal Society, Interface 7: 905–919 doi:10.1098/rsif.2009.0400. 10.1098/rsif.2009.0400 PMC2871802 19940002

[27] Christensen N, Bounds C (2010) Cross-protective responses to human papillomavirus infection. Future Virology 5: 163.

[28] Booth M, Graham A (2008) Parasitic co-infections: challenges and solutions. Parasitology 135: 749 doi:10.1017/S0031182008000413. 18578954 10.1017/S0031182008000413

[29] Tilman D (1982) Resource competition and community structure. Volume 17. Princeton University Press. p. 7162524

[30] Simen-Kapeu A, Kataja V, Yliskoski M, Syrjänen K, Dillner J, et al (2008) Smoking impairs human papillomavirus (HPV) type 16 and 18 capsids antibody response following natural HPV infection. Scandinavian Journal of Infectious Diseases 40: 745–751. 19086247 10.1080/00365540801995360

[31] Luchters SMF, Vanden Broeck D, Chersich MF, Nel A, Delva W, et al (2010) Association of HIV infection with distribution and viral load of HPV types in Kenya: a survey with 820 female sex workers. BMC infectious diseases 10: 18 doi:10.1186/1471-2334-10-18. 20102630 10.1186/1471-2334-10-18 PMC2845133

[32] McKenzie ND, Kobetz EN, Hnatyszyn J, Twiggs LB, Lucci JA 3rd (2010) Women with HIV are more commonly infected with non-16 and -18 high-risk HPV types. Gynecologic oncology 116: 572–577 doi:10.1016/j.ygyno.2009.10.058. 19906410 10.1016/j.ygyno.2009.10.058

[33] Lafferty KD, Allesina S, Arim M, Briggs CJ, De Leo G, et al (2008) Parasites in food webs: the ultimate missing links. Ecology letters 11: 533–546 doi:10.1111/j.1461-0248.2008.01174.x. 18462196 10.1111/j.1461-0248.2008.01174.x PMC2408649

[34] Grenfell B, Harwood J (1997) (Meta) population dynamics of infectious diseases. Trends in Ecology & Evolution 12: 395–399. 21238122 10.1016/s0169-5347(97)01174-9

[35] Mideo N, Alizon S, Day T (2008) Linking within- and between-host dynamics in the evolutionary epidemiology of infectious diseases. Trends in ecology & evolution 23: 511–517 doi:10.1016/j.tree.2008.05.009. 18657880 10.1016/j.tree.2008.05.009

[36] Merikukka M, Kaasila M, Namujju PB, Palmroth J, Kirnbauer R, et al (2011) Differences in incidence and co-occurrence of vaccine and nonvaccine human papillomavirus types in Finnish population before human papillomavirus mass vaccination suggest competitive advantage for HPV33. International journal of cancer 128: 1114–1119 doi:10.1002/ijc.25675. 20839258 10.1002/ijc.25675

[37] Tota J, Agnihotram RV, Coutlée F, Villa LL, Richardson H, et al.. (2011) Epidemiologic approach to evaluate potential for HPV type replacement post-vaccination: O–01.04.

[38] García-Vallvé S, Alonso A, Bravo IG (2005) Papillomaviruses: different genes have different histories. Trends in microbiology 13: 514–521 doi:10.1016/j.tim.2005.09.003. 16181783 10.1016/j.tim.2005.09.003

[39] Doorbar J (2005) The papillomavirus life cycle. Journal of clinical virology 32 Suppl 1S7–15 doi:10.1016/j.jcv.2004.12.006. 15753007 10.1016/j.jcv.2004.12.006

[40] Bauer AL, Beauchemin C a a, Perelson AS (2009) Agent-based modeling of host–pathogen systems: The successes and challenges. Information Sciences 179: 1379–1389 doi:10.1016/j.ins.2008.11.012. 20161146 10.1016/j.ins.2008.11.012 PMC2731970

[41] Funk GA, Jansen VA, Bonhoeffer S, Killingback T (2005) Spatial models of virus-immune dynamics. Journal of theoretical biology 233: 221–236 doi:10.1016/j.jtbi.2004.10.004. 15619362 10.1016/j.jtbi.2004.10.004

[42] Vaccarella S, Franceschi S, Herrero R, Schiffman M, Rodriguez AC, et al (2011) Clustering of multiple human papillomavirus infections in women from a population-based study in Guanacaste, Costa Rica. The Journal of infectious diseases 204: 385–390 doi:10.1093/infdis/jir286. 21742837 10.1093/infdis/jir286 PMC3132145

[43] Quint W, Jenkins D, Molijn A, Struijk L, van de Sandt M, et al (2012) One virus, one lesion–individual components of CIN lesions contain a specific HPV type. The Journal of pathology 227: 62–71 doi:10.1002/path.3970. 22127961 10.1002/path.3970

[44] Wodarz D, Levy DN (2009) Multiple HIV-1 infection of cells and the evolutionary dynamics of Cytotoxic T Lymphocyte escape mutants. Evolution: 2326–2339. doi:10.1111/j.1558-5646.2009.00727.x. 10.1111/j.1558-5646.2009.00727.x 19486149

[45] Polis G, Myers C, Holt R (1989) The ecology and evolution of intraguild predation: potential competitors that eat each other. Annual Review of Ecology and Systematics 20: 297–330.

[46] Antia R, Bergstrom CT, Pilyugin SS, Kaech SM (2003) Models of CD8+ Responses: 1. What is the Antigen-independent Proliferation Program. Journal of Theoretical Biology 221: 585–598. 12713942 10.1006/jtbi.2003.3208

[47] Nowak M, Bangham CRM (1996) Population dynamics of immune responses to persistent viruses. Science 272: 74–79. 8600540 10.1126/science.272.5258.74

[48] Liang H, Miao H, Wu H (2010) Estimation of Constant and Time-Varying Dynamic Parameters of Hiv Infection in a Nonlinear Differential Equation Model. The annals of applied statistics 4: 460–483. 20556240 10.1214/09-AOAS290 PMC2885820

[49] Wang H-K, Duffy AA, Broker TR, Chow LT (2009) Robust production and passaging of infectious HPV in squamous epithelium of primary human keratinocytes. Genes & Development 23: 181–194. 19131434 10.1101/gad.1735109 PMC2648537

[50] Stanely MA (2006) Immunobiology of Papillomaviruses. In: Campo MS, editor. Papillomavirus Research: from natural history to vaccines and beyond. Horizon Scientific Press. p. 311.

[51] Culp TD, Christensen ND (2004) Kinetics of in vitro adsorption and entry of papillomavirus virions. Virology 319: 152–161 doi:10.1016/j.virol.2003.11.004. 14967496 10.1016/j.virol.2003.11.004

[52] Frattini MG, Lim HB, Laimins LA (1996) In vitro synthesis of oncogenic human papillomaviruses requires episomal genomes for differentiation-dependent late expression. PNAS 93: 3062–3067. 8610168 10.1073/pnas.93.7.3062 PMC39761

[53] Althaus CL, De Boer RJ (2011) Implications of CTL-mediated killing of HIV-infected cells during the non-productive stage of infection. PloS ONE 6: e16468 doi:10.1371/journal.pone.0016468.g002. 21326882 10.1371/journal.pone.0016468 PMC3034731

[54] Adams BM, Banks HT, Davidian M, Kwon H-D, Tran HT, et al (2005) HIV dynamics: Modeling, data analysis, and optimal treatment protocols. Journal of Computational and Applied Mathematics 184: 10–49 doi:10.1016/j.cam.2005.02.004.

[55] de Boer R, Oprea M, Antia R, Murali-krishna K, Ahmed R, et al (2001) Recruitment times, proliferation, and apoptosis rates during the CD8+ T-cell response to lymphocytic choriomeningitis virus. Journal of Virology 35: 10663–10669 doi:10.1128/JVI.75.22.10663-10669.2001. 10.1128/JVI.75.22.10663-10669.2001 PMC114648 11602708

[56] Ribeiro RM, Mohri H, Ho DD, Perelson AS (2002) In vivo dynamics of T cell activation, proliferation, and death in HIV-1 infection: why are CD4+ but not CD8+ T cells depleted? PNAS 99: 15572–15577 doi:10.1073/pnas.242358099. 12434018 10.1073/pnas.242358099 PMC137758

[57] Stämpfli MR, Anderson GP (2009) How cigarette smoke skews immune responses to promote infection, lung disease and cancer. Nature reviews Immunology 9: 377–384. 10.1038/nri2530 19330016

[58] Xi LF, Koutsky LA, Castle PE, Edelstein ZR, Meyers C, et al (2009) Relationship between cigarette smoking and human papilloma virus types 16 and 18 DNA load. Cancer epidemiology, biomarkers & prevention 18: 3490–3496. 10.1158/1055-9965.EPI-09-0763 PMC2920639 19959700

[59] Nadais RDF, Campaner AB, Piato S, Longo Galvão MA, dos Santos RE, et al (2006) Langerhans’ cells and smoking in intraepithelial neoplasia of the cervix. Gynecologic oncology 102: 356–360 doi:10.1016/j.ygyno.2005.12.030. 16472845 10.1016/j.ygyno.2005.12.030

[60] Schiller JT, Castellsagué X, Villa LL, Hildesheim A (2008) An update of prophylactic human papillomavirus L1 virus-like particle vaccine clinical trial results. Vaccine 26 Suppl 1K53–61 doi:10.1016/j.vaccine.2008.06.002. 18847557 10.1016/j.vaccine.2008.06.002 PMC2631230

