# Supplementary figures and images for: Food Webs in the Human Body: Linking Ecological Theory to Viral Dynamics
Source: PLoS One. 2012 Nov 14;7(11):e48812. doi: 10.1371/journal.pone.0048812 (PMC3498237; doi:10.1371/journal.pone.0048812)

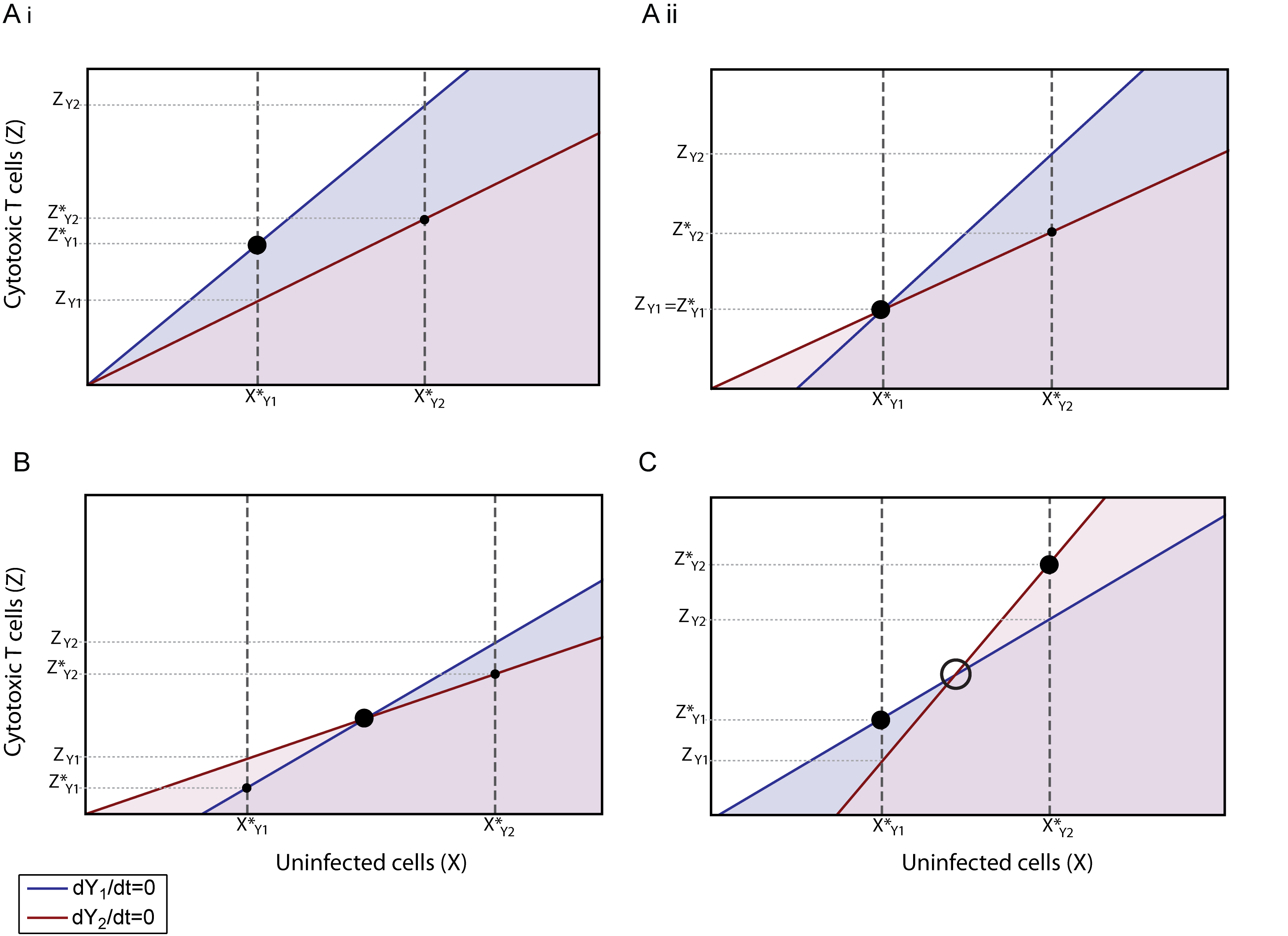

Supplement: Figure S1 — Phase-plane cases. A: Competitive exclusion. (i and ii) The black dots represent the equilibrium solution of each subsystem (i.e. where X, Yi, Z can exist together). The winner is determined by invasion criteria, such that if the isocline of strain i is above the equilibrium of the subsystem with strain j, then strain i can invade but strain j cannot. Strain i then, is the winner and its subsystem equilibrium is an attractor. B: Coexistence. Here the isoclines cross inside the two subsystem curves and both strains can invade, thus the interior equilibrium is stable. Finally, C: Priority Effects. The interior equilibrium is unstable therefore the initial conditions determine who wins. (TIF) [file pone.0048812.s001.tif]

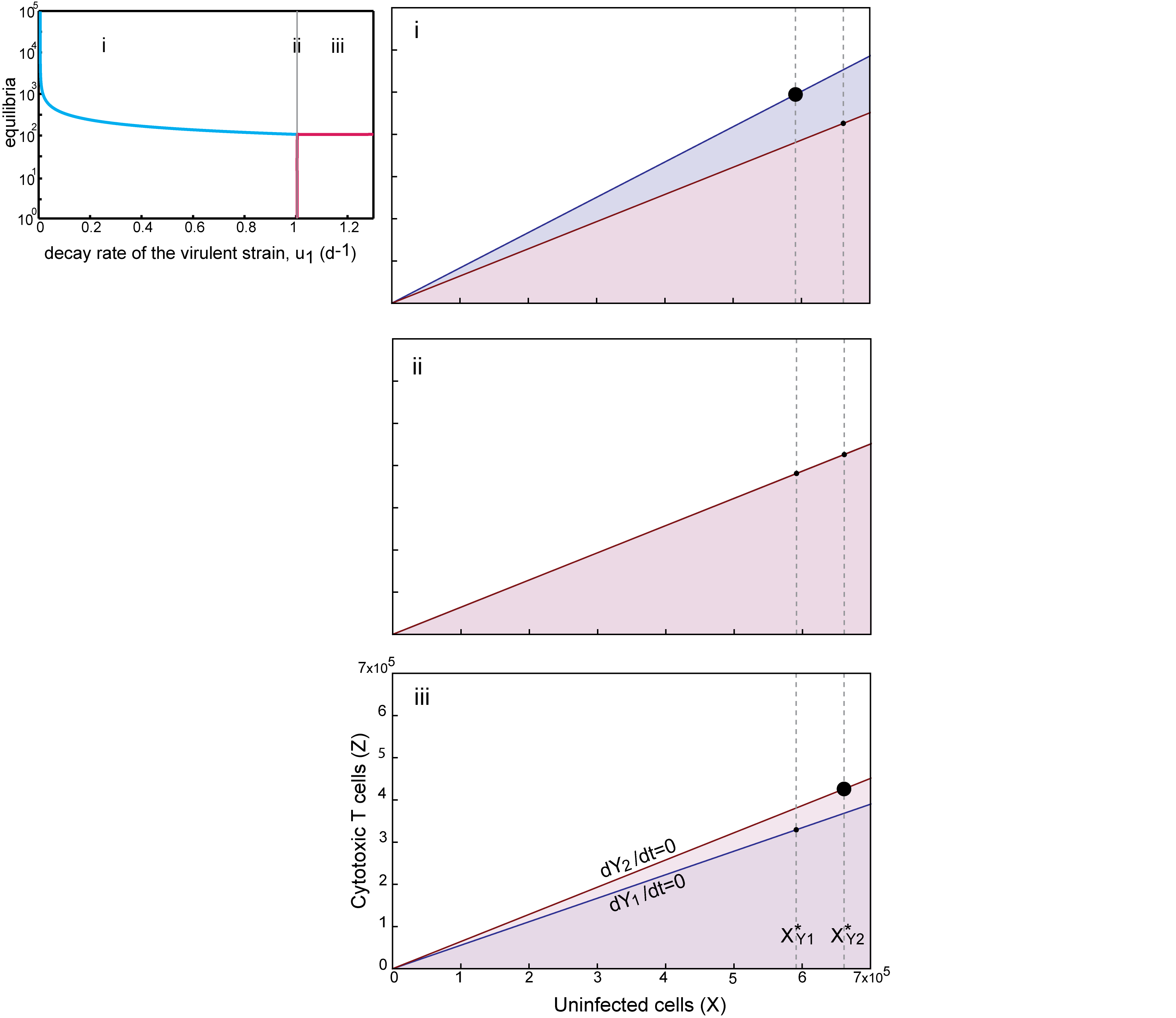

Supplement: Figure S2 — The reproduction and decay trade-off in HPV with matching phase-planes. No coexistence. Plots (i) and (iii) represent the phase-planes before and after the bifurcation, respectively, and plot (ii) is at the bifurcation. Parameter estimates. HPV: λ = 36000 cells•day−1 [49], d = 0.048 day−1 [50], β1 = β1 = 0.0067 day−1 [51], k1 = 100 virions•cell−1•day−1 , k2 = 50 virions•cell−1•day−1 [52], u2 = 0.52 day−1 [23], and since HPV is a non-lytic virus d = a1 = a2 = 0.048 day−1 [50]. Immunity: p1 = p2 = 1 day−1 [53], m = 0.01 day−1 [56], c1 = c2 = 0.1 day−1 [44] (TIF) [file pone.0048812.s002.tif]

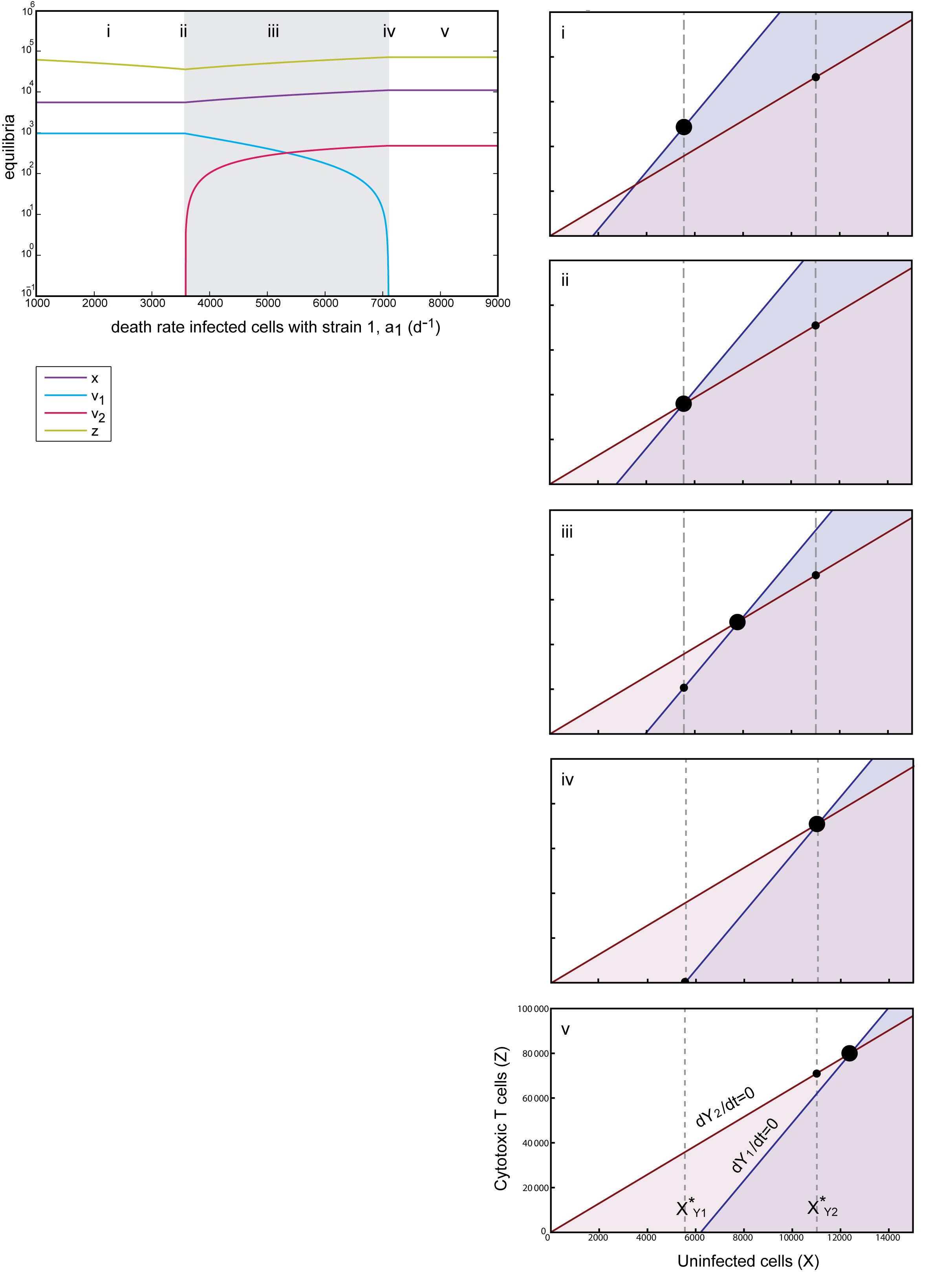

Supplement: Figure S3 — The reproduction and lytic effect trade-off in HPV allows for coexistence. Plots (i) and (v) are before and after the bifurcations, (ii) and (iv) are at the bifurcations, and (iii) is stable coexistence. Parameter estimates. HPV: λ = 36000 cells•day−1 [49], d = 0.048 day−1 [50], β1 = β1 = 0.0067 day−1 [51], k1 = 100 virions•cell−1•day−1 , k2 = 50 virions•cell−1•day−1 [52], u2 = 0.52 day−1 [23], and since HPV is a non-lytic virus d = a1 = a2 = 0.048 day−1 [50]. Immunity: p1 = p2 = 1 day−1 [53], c1 = c2 = 0.1 day−1 [44], m = 0.5 day−1 [55]. (TIF) [file pone.0048812.s003.tif]
