# Supplementary material for: Food Webs in the Human Body: Linking Ecological Theory to Viral Dynamics
Source: PLoS One. 2012 Nov 14;7(11):e48812. doi: 10.1371/journal.pone.0048812 (PMC3498237; doi:10.1371/journal.pone.0048812)
Supplement: Text S1 — Conditions for Coexistence inside a Host. (PDF) [file pone.0048812.s004.pdf]

Supporting Information

Conditions for Coexistence inside a Host

Some pathogens are rarely found to coinfect a single host, while others are regularly found together. Here we see that mutualism or facilitation is not necessary to explain the coexistence of strains inside the same host. Instead we show that community ecology concepts such as in-host trade-offs and enrichment greatly influence whether coexistence is possible inside a host.

We investigated the conditions necessary for coexistence in a two strain community with asymmetric immunity attack (i.e. cross-reactivity) and asymmetric resource competition. Following Holt et al. (1994), we developed graphical invasion criteria for the model system (model 2). Specifically, we looked at the conditions that allow either strain (starting from a low density) to invade the X- Y_i -Z sub-system containing the other strain at equilibrium (e.g. whether Y_2 can invade X-Y_1-Z). To accomplish this and to simultaneously create graphical conditions for coexistence consistent with the approach of Holt et al. (1994) [1], we looked at informative two-dimensional reduced nullsurfaces for the cases X-Y_1-Z and X-Y_2-Z.

To reduce the nullsurfaces, first recognize that for either X-Y_i-Z sub-system, its $dZ/dt = 0$ isocline completely determines the Y_i * equilibrium value (i.e. when $dZ/dt = 0$, then $Y_i * = (m/c_i)$). Substituting this value into $dX/dt = 0$ and solving yields;

$$X_{Yi}* = \lambda\, c_i\, u_i \;/\; (u_i\;\; d\;\; c_i + \beta_i\;\, k_i\;\; m), \qquad \text{(S.1)}$$

Which are the invariant reduced nullsurface (Fig. S.1). Next we solved for the $dY_i/dt = 0$ nullsurfaces;

$$Z_{Yi}* = X_{Yi}*\, (k_i\;\beta_i\;\; / \;u_i\;\; p_i)\;\; - a_i \;/\; p_{i,} \qquad \text{(S.2)}$$

which are identified graphically in Fig. S1. Taken together, this produces a two-dimensional plot of reduced nullsurfaces that represent the underlying three-dimensional nullsurfaces for each sub-system (X-Y_i-Z). The intersection of the two reduced nullsurfaces ($dX/dt = 0$ and $dY_i/dt = 0$) give us the X-equilibrium value and the Z-equilibrium value for the specific three-dimensional sub-system. In a sense, the substitution of the Y_i*-equilibrium allows us to lose a dimension at the cost of not showing the exact value of the Y_i * (although we know it is $Y_i^* = (m/c_i)$).

To complete the invasion analysis, we need to consider the dynamics of the two possible invasion cases. Can Y_j invade (allowing Y_j to be an arbitrarily small number, ε) when the system is at the X-Y_i-Z sub-system equilibrium (for j = 1,2 and i = 1,2) ? Graphically, the invasion criteria for Y_j occurs when the $dY_j/dt = 0$ reduced nullsurface lies above the intersection of the $dX/dt = 0$ (eq. S.1; for X-Y_i-Z sub-system) and the $dY_i/dt = 0$ (i.e. lies above the equilibrium for the X-Y_i-Z sub-system). The above conditions ensure that the growth of Y_j is positive. We can classify the fate of the invading Y_j, given X-Y_i-Z, into the four possible cases:

(1) strain j invades and excludes strain i (Fig. S1 A i and ii)

if $Z_{Yi}^* < Z_{Yj}$ and $Z_{Yj}^* \geq Z_{Yi}$

(2) both strains can invade, i.e. coexistence (Fig. S1 B)

if $Z_{Yi}^* < Z_{Yj}$ and $Z_{Yj}^* < Z_{Yi}$

(3) neither can invade the other, i.e. priority effects (Fig. S1 C)

if $Z_{Yi}^* > Z_{Yj}$ and $Z_{Yj}^* > Z_{Yi}$

(4) neutral, i.e. coexistence is not possible (Fig. S2 ii)

if $Z_{Yi}^* = Z_{Yj}$ and $Z_{Yj}^* = Z_{Yi}$

where Z_{Yi}^* is the intercept of $dY_i/dt = 0$ (eq.S.2) of the X-Y_i-Z sub-system with $dX/dt = 0$ (eq. S.1) of the same subsystem system,

$Z_{Yi}* = \lambda c_i k_i \beta_i / (p_i (u_i d c_i + \beta_i k_i m)) - a_i / p_{i,}$,

and where Z_{Yi} is the intercept of $dY_i/dt = 0$ (eq.S.2) of the X-Y_i-Z sub-system with $dX/dt = 0$ (eq. S.1) of the other subsystem, X-Y_j-Z,

$Z_{Yi} = \lambda c_j u_j k_i \beta_i / (u_i p_i (u_j d c_j + \beta_j k_j m)) - a_i / p_i$.

Note that $Z_{Yj}*$ and Z_{Yj} are found in a likewise fashion.

Case (2) above depicts coexistence since both strains can invade when rare, and the boundary sub-system repels into the four-dimensional system yielding an interior solution (Fig. S1 B).

To illustrate these ideas more clearly, we show how these nullsurfaces match specific regions of their corresponding bifurcation plot (Fig. S2 and S3). For example, the reproduction and decay trade-off falls into case (4) above, which drives an instantaneous switching from one exclusion region to the other (see Fig. S2). This can also be appreciated in the equations (model 2) because, from looking at the dY_i/dt equations, we can see that changes to either the replication rate, k_i, or to the decay rate, u_i, only affect β_i' (they are both inside β_i') therefore the strains can only differ from one another by β_i', while their $dY_i/dt = 0$ Z-axis intercepts remain identical, $a_1/p_1 = a_2/p_2$ (eq. S.2). This implies, for instance, that increasing u_1 only changes the slope of $dY_1/dt = 0$ until, and past, the point where the two lines completely overlap, driving a degenerate bifurcation (Fig.S2 ii). Biologically then, this trade-off changes which strain excludes the other and does not allow for coexistence (technically a coexistence region exists but it is infinitesimally small).

In Figure S3 we give an example of a trade-off that can give coexistence. Consider that increased replication rates of the virus, high k, could have a negative effect on the lifespan of its host cell (causing an increase in a). Though viruses can be lytic (virus kills the host cell) or non-

lytic (the average life span of the host cell is not affected by viral infection), there should still be some variation in lytic effect (the rate at which the virus kills the host cell) and it would be interesting to study whether rapidly replicating strains, on average, kill their host cells faster than other strains. We considered this hypothetical in-host trade-off and found that it allowed for coexistence, albeit for parameter values of the infected cell death rate, *a*, that were not biologically feasible. This lack of biological realism, however, depends on the parameter estimates of the attack rates of the CTL, *p*, which, to our knowledge, is currently not well measured. Once again, better estimates of the timing of vital processes happening during infections could help improve our ability to understand when these in-host trade-offs could be affecting the community dynamics of the infection.

There are other factors that can affect whether or not coexistence is possible. For example, we know from ecology that resource availability and resource use strategies are important. Here, we assumed that the resource, the uninfected cell population, grows at a constant rate, λ. This may not be true, depending on the cell type. Also, not all cells are necessarily accessible at all times and, the growth rates of the cells could range considerably, again, depending on the target cell type. In reference to the latter, we found that changing λ from low to high values, had the singular effect of moving $X_{Yi}*$ along the *X*-axis either towards the origin when small or away from the origin when λ was large (not shown). Thus, depending on the movement of the $dY_i/dt = 0$ nullsurfaces caused by changing the bifurcation parameter of interest, the change in λ affects the likelihood of coexistence. If the movement of the nullsurfaces is such that they cross more easily close to the origin, then if the $X_{Yi}*$ are near the origin then it is more likely that small changes in the bifurcation parameter will lead to them crossing within the bounded region. Also note, that lower λ values shrank these bounded regions and could also

change (i.e. decrease) the value of the bifurcation parameter. These changes can be particularly important for keeping the values of the bifurcation parameter within a biologically feasible range. This highlights that the concept of “enrichment” from ecology should be investigated further in these systems. Whether the “paradox of enrichment” is found or not, infectious disease dynamics studies could benefit from developing their own version of enrichment theory.

Finally, the linear rates used in these equations are the simplest assumptions possible and thus give simple straight nullsurfaces. With the inclusion of carrying capacities or more realistic functional responses the shapes of the nullsurfaces would be more interesting and could make coexistence more probable.

References

1. Holt RD, Grover J, Tilman D (1994) Simple rules for interspecific dominance in systems with exploitative and apparent competition. American Naturalist 144: 741–771.
