# Supplementary material for: Food Webs in the Human Body: Linking Ecological Theory to Viral Dynamics
Source: PLoS One. 2012 Nov 14;7(11):e48812. doi: 10.1371/journal.pone.0048812 (PMC3498237; doi:10.1371/journal.pone.0048812)
Supplement: Box S1 — Using modules to understand community dynamics. (PDF) [file pone.0048812.s005.pdf]

Box S1. Using modules to understand community dynamics

The theory of food web modules allows us to determine the conditions that lead to ecological outcomes such as dominance, coexistence and exclusion. For example, competitive exclusion during resource competition is determined by the R* rule: the species that can maintain a viable population (birth rate is equivalent to death rate; A below) at the lowest density of the shared resource will drive its competitor to extinction [1]. Competitive exclusion can also happen due to indirect competition via a shared predator, as in apparent competition. A similar rule, the P* rule, governs the outcome of this module and it states that the species that can withstand the highest levels of predation will exclude its competitor [2]. Importantly, exclusion is not the only outcome of these communities. Understanding how species coexist has been a focus of ecologists for decades [34, 35], and modular theory has helped decipher this as well.

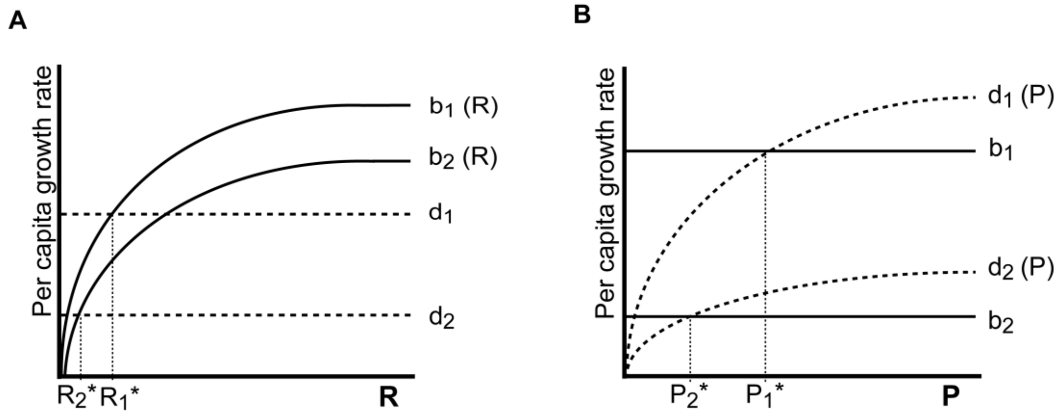

Adapted from Chase and Leibold (2003) Fig. 2.3[5]. A: R* rule; species 2 wins (lowest R*). B: P* rule; species 1 wins (highest P*). Shared resource (R), predator (P), birth rate (bi), and death rate of species i (di). Intersection of birth rate and death rate gives R* or P* for a species.

The relative strengths of the interactions in communities determines whether coexistence or exclusion will result and thus if these rule apply. For example, in the diamond module, where apparent and resource competition exist jointly, whether there is symmetric predation (strength of predation has equal effect on both species) or symmetric resource competition, then the outcomes are determined by the R* or the P* rule respectively [6]. For example, if predation on one competitor is stronger than on the other but resource competition is symmetric in strength, then the system dynamically collapses into the apparent competition module, and the qualitative outcome depends on the P* rule. Thus in this case, the outcome of this diamond module is determined by a simple rule that comes from a simpler imbedded module (apparent competition).

The mathematical forms of consumer-resource models and in-host dynamic models are very similar, and not surprisingly the P* and R* rules have been found to also apply to strain interactions, when strains are simultaneously influenced by competition (e.g. for cells) and immunity attack [7]. However, new rules specific to in-host systems should be sought after. Also, finding relative interactions strengths is lacking attention in in-host studies, but given their explanatory power in ecological theory their potential to elucidate in-host dynamical outcomes they are an exciting avenue for in-host ecology and should be explored further.

References

1. Tilman D (1982) Resource competition and community structure. Volume 17. Princeton University Press. p.

2. Holt RD (1977) Predation, apparent competition, and the structure of prey communities. Theoretical Population Biology 12: 197–229.

3. Sommer U, Worm B, editors (2002) Competition and Coexistence. Verlag Berlin Heidelberg: Springer. p.

4. Tokeshi M (1999) Species Coexistence: ecological and evolutionary perspectives. Oxford: Blackwell Sciences Ltd. p.

5. Chase JM, Leibold MA (2003) Ecological Niches: classical and contemporary approaches. Chicago: The University of Chicago Press. p.

6. Holt RD, Grover J, Tilman D (1994) Simple rules for interspecific dominance in systems with exploitative and apparent competition. American Naturalist 144: 741–771.

7. Mideo N (2009) Parasite adaptations to within-host competition. Trends in parasitology 25: 261–268.
